# Social effects of HIV disclosure, an ongoing challenge in young adults living with perinatal HIV: a qualitative study

**DOI:** 10.3389/fpubh.2023.1150419

**Published:** 2023-05-19

**Authors:** Linda Aurpibul, Arunrat Tangmunkongvorakul, Chanidapa Detsakunathiwatchara, Supunnee Masurin, Angkana Srita, Patcharaporn Meeart, Walailak Chueakong

**Affiliations:** Research Institute for Health Sciences, Chiang Mai University, Chiang Mai, Thailand

**Keywords:** HIV disclosure, social effects, perinatal HIV infection, adolescents, young adults

## Abstract

**Introduction:**

Young adults with perinatal HIV (YAPHIV) have survived the long journey of life while living with HIV since early childhood. We explore the HIV disclosure experience and its social effects from their perspectives.

**Methods:**

The qualitative study was conducted from June to November 2022 in Chiang Mai, Thailand. Data were collected through individual in-depth semi-structured interviews with 20 YAPHIV at the median age of 25 years. Content analysis was used to identify themes from the interview transcripts.

**Results:**

Most participants learned their HIV status from their parents, caregivers, healthcare providers, or other people in community during their childhood. Some were disclosed later in adolescent years. HIV disclosure to others was associated with various experiences in different stages of life. While some YAPHIV decided not to disclose their HIV status to anyone, it also had social effects. Three major themes were identified: (1) positive social effects of HIV disclosure (perceived social acceptance, perceived social support); (2) negative social effects of HIV disclosure (effects on child rearing, schooling, and family relationship); and (3) HIV non-disclosure (anticipated stigma, negative effects on the quality of employment, and relationships). An emerging theme was a need for peer support mentioned by several YAPHIV as they would like to discuss with somebody and share their feelings while living with HIV.

**Conclusion:**

HIV disclosure remains challenging for YAPHIV while growing up and moving toward adult milestones. Better understanding their situations and perspectives would allow healthcare providers to provide them with updated HIV knowledge, coping skills, and psychosocial support.

## Introduction

According to the UNAIDS, globally the estimated total number of young people living with HIV in 2019 was 3.4 million (1). This included youth with behaviorally acquired HIV infection and those with perinatal HIV (PHIV). In Thailand, many children with PHIV who received antiretroviral treatment (ART) have grown up into their adolescence and adulthood (2). They needed to continue ART while transitioning from pediatric to adult services and thereafter. Some had comorbidities or complications (3, 4). Reports from multiple countries worldwide showed that youth with PHIV struggled with the transition process, and post-transition outcomes were not always favorable (5). Suboptimal ART adherence in adolescents and young adults was associated with virologic non-suppression, revisiting of opportunistic infections, and AIDS-related mortality (6). It is of particular concern that sexually active youth with PHIV on ART who had unsustainable virologic suppression might transmit HIV (7), especially when the partners do not know their serostatus and miss opportunities to protect themselves by using either condom or pre-exposure prophylaxis (8).

HIV disclosure is a known important determinant of both physical and mental individual health outcomes, which remains challenging for individuals living with HIV at any age (9). In the life of children with PHIV, the first disclosure journey started when they were made aware of their serostatus. According to the World Health Organization (WHO), children of school age should be informed about their HIV status between 6 and 12 years (10). Although the benefits of the disclosure on adherence, physical health, and sexual behavior responsibility of youth have been confirmed (11), the rate of HIV disclosure to children varies from one setting to another. Disclosure of HIV status to children is a difficult task for parents/caregivers, as it is emotive and can be associated with either positive or negative health outcomes. Several factors were identified by caregivers who deferred disclosure to their child, including the concerns about the child’s negative reaction, and inappropriate disclosure to others which might bring about HIV-related stigma and discrimination (12). Some postponed the disclosure until the child reached their adolescent years. However, at least one study documented an association between non-disclosure and virologic non-suppression (13).

When children become adolescents, they need to expose themselves to broader social networks and communities. Self-disclosure refers to the autonomous revelation of one’s HIV status to sexual partners, family members/relatives, or other persons in community (14). It might be beneficial to the disclosers to receive social support (15). Meanwhile, it can bring about negative consequences including HIV-related stigma and discrimination (16). In adolescents with PHIV, disclosure to friends is difficult for them due to rejection sensitivity during this transitional period of life (17). A Thai study revealed that only 22% disclosed their status to friends while 48% did not disclose to anyone (18). In young adult life, self-disclosure of their serostatus to sexual partners is critical as it is related to HIV transmission. There are various reasons for non-disclosure including the overwhelming self-stigma and anticipated negative social effects.

Currently in Thailand, many young adults with perinatal HIV (YAPHIV) have survived entering the third decade of life. The study aimed to explore their HIV disclosure experience and its social effects to allow more understanding of what was going on in the real world. Greater awareness of this issue could help guide the development of interventions, programs, or policy to support the process and enhance disclosure self-efficacy, with the goal to maximize individual health benefits and to minimize the negative consequences.

## Methods

### Study population

A qualitative study was conducted at the Research Institute for Health Sciences, Chiang Mai University, in northern Thailand from to June to November 2022. The inclusion criteria were (1) aged 20–30 years, (2) living with perinatal HIV, (3) disclosed to their own HIV status either in childhood (before age 12) or adolescence (13–17 years), and (4) willing to join the study and share their experiences. Participants were recruited by outreach workers and health staff who were familiar with them since they previously attended local pediatric HIV clinics. Purposive sampling techniques were used. After the initial telephone contact, prospective participants were asked to come to the study site where they were informed about the purpose of the study. Written informed consent was obtained from those who agreed to participate prior to the in-depth interview session. The targeted number of participants of 20–25 was estimated to reach data saturation (19).

### Data collection

The study activities were conducted in a private room in the research clinic at the Research Institute for Health Sciences, Chiang Mai University. Demographic information collected included age, sex, highest level of education, and current occupation. The qualitative in-depth interviews were conducted by a research team, all of whom are females: LA is a pediatrician, CD, SUM, and ANS are clinical research nurses, PM and WC are research assistants, and AT is a behavioral sciences researcher. The team was trained and coached by AT who is an expert qualitative researcher. During each interview session, only an interviewer and the participant were present. Each interview commenced with a brief self-introduction by the researcher. An interview guide was used to facilitate conversation around the participant’s experiences on HIV disclosure and its social effects in their life. This interview guide was developed by the study team based on a thorough literature review and pilot testing. All interviews were recorded, and the participants were informed about the audio recording. The individual in-depth semi-structured interviews were conducted with each participant only once. Field notes were made by each interviewer after the interview ended. The transcripts were discussed and reviewed during the monthly researcher meeting.

### Data analysis

Qualitative data from IDI audio files were transcribed and reviewed by leading researchers (LA and AT). We analyzed transcribed data in Dedoose Software [Dedoose Version 9.0.90, a web application for managing, analyzing, and presenting qualitative and mixed method research data (2023). Los Angeles, CA: SocioCultural Research Consultants, LLC].^1^ We explored how HIV disclosure impacts individual depending on whether disclosure happened in childhood, adolescence, or adulthood, using a content analysis approach (20). Two researchers reviewed all transcripts to determine the presence of themes. We developed a codebook with coding rules and independently coded the data for each transcript. We discussed and refined the codebook to understand our findings and the meaning of the contents. All transcripts were in Thai; we only translated selected quotes into English during manuscript preparation to support the results in each theme.

### Ethics consideration

The study was approved by the Human Experimentation Committee, Office of Research Ethics of the Research Institute for Health Sciences, Chiang Mai University (certificate of approval no. 21/2022).

## Results

A total of 20 YAPHIV participated in the study, including 8 females and 12 males. Their median age was 25 years (interquartile range 23–28). The highest levels of education completed were vocational school (*N* = 10), high school (*N* = 3), middle school (*N* = 5), and primary school (*N* = 2). Regarding employment, 18 worked full-time, one was studying and working part-time, and another one was seeking a job. In terms of marital status, 14 were single, 5 were coupled, and 1 was separated. All were currently receiving ART and HIV care at public hospitals in Chiang Mai under the National AIDS program. In the three periods of life, childhood (before age 12), adolescence (13–17 years), and adulthood (≥18 years), the analysis revealed three main themes: positive and negative various social effects of HIV disclosure and the presence of non-disclosure. The need for peer support was an emerging theme associated with HIV disclosure in adulthood period.

### Childhood

HIV disclosure occurred around the age of 10 in 14 YAPHIV. In early childhood, many participants were not yet aware of their HIV status. Most families did not conduct formal HIV disclosure to children. Some participants learned it by themselves while living with others in the villages, while their parents, caregivers, or healthcare providers started talking about HIV later in their early adolescent years. The participants provided little experience of HIV disclosure-related trouble during childhood. From the in-depth interviews, we learned that most of them resided in rural villages from 1995 to 2000, when AIDS deaths were prevalent in Northern Thailand. At that time, no HIV treatment was available. One or both biological parents of many participants died of AIDS, and everyone in the community knew their family serostatus. HIV disclosure was not a challenge for most participants during their childhood.

### Positive social effects of HIV disclosure

#### Perceived social acceptance

Participants stated that they could live normally like others while receiving antiretroviral treatment and living in the supportive environment of parents or relatives in extended families. A male participant described his self-acceptance and family situation from his childhood memory. His friends accepted him as a part of the group. Although they used separate personal items while eating together, it was a general practice, and he did not feel like others were discriminating against him.

*“They all knew that my mom and dad have HIV, and I also have it. We led a normal life like others in the village. Dad was the one who earned money for our family. He worked in a chain food store. For my friends who hung out together, we shared food and drinks. Surely, we used separated plates and utensils on the same table.”* (B041, male 21–24 years)

Two other participants revealed their feelings when they first learned their HIV status as neutral. They could accept it as a part of their ordinary life and did not experience any social effects related to HIV disclosure. With self-acceptance, their life could go on.

*“I started syrup medicine at the age of 4. At that time, I did not know what it was for, but I did love its sweetness. My mom told me about HIV when I was in grade 2, and nothing changed in my life. All my family members and relatives knew my status, and we stayed together with no problem.”* (B043, female 21–24 years)

*“I was aware of my HIV status when I overheard some adult neighbors’ conversation. I went home and asked my grandpa and grandma if it was true, and they said “yes”. I did not feel anything. My life continued as before.”* (B044, male 25–29 years)

#### Perceived social support

Many YAPHIV felt they perceived support from family and community members in their childhood around the time their HIV status was disclosed to them. During school age, for some, everyone in school knew their HIV status. There was no profound discrimination that they could feel as a child. A male participant claimed that he was over-protected by a teacher in school after she knew his HIV status. Her behavior attracted others’ attention to him, but it did not affect his life. He felt beloved by everyone.

*“When I was in grade 3–4, my friends at school knew [my HIV status] because a teacher wanted to protect me. She told everyone not to play rough with me as I was not well. Living with my uncle and aunt, I felt very healthy. My classmates played with me like normal. I think some neighbors also knew it, but no one treated me badly.”* (B055, male 25–29 years)

Some participants were not tested for HIV until they became ill and needed medical care. A female participant described her disclosure experience during grade school. She said that she was sad after learning about her HIV status from her grandma, but that she received mental support from her aunt.

*“I learned my HIV at the age of 10 from my grandma who was the one who accompanied me to the hospital. I felt down and kept thinking “why me?”, “why do I have it, and others don’t?”. My aunt told me not to be worried. She said that the disease was treatable.”* (B058, female 21–24 years)

### Negative social effects of HIV disclosure

Effect on child rearing was described by a female participant who grew up with her stepmother. Back then, without widely accessible ART, most children with HIV could not live long and would die soon after the diagnosis. She started ART at a pediatric HIV clinic in a large hospital after becoming ill and being hospitalized before age 10. Her father focused on his work as a government officer, leaving child rearing to his new wife. The participant was small for her age. Although she became healthier after the treatment initiation, she still needed to take medication every day. When her serostatus became known to her stepmother, who saw her taking the pills, she forced the girl to leave school to stay home and do housework and look after her infant stepsister.

“*It was a painful memory that I want to forget. My stepmom was so cruel, she hit me every day. One day she told me to stop going to school, because I did not have to study anymore due to having HIV. She forced me to stay home to look after the baby and to work in her laundry service. All my neighbors knew, and they tried to rescue me.*” (B054, female 21–24 years)

### HIV non-disclosure

We found that not all participants decided to socially disclose. A female participant shared that she kept her HIV status a secret from all her relatives. Only her mom (who also received ART) knew. Her mom supported her adherence since the start of treatment, while her grandparents, with whom she lived, were not aware of her serostatus at all. When the interviewer probed about the reason for non-disclosure, it seemed like there was an issue of anticipated stigma and fear of discrimination as she described her negative impression when seeing what happened to other people who disclosed their HIV status in the community.

*“When I started my meds at the age of 6, I lived with my grandparents. They did not know my serostatus. My mom worked in another province. Only mom and I knew it, and she was the one who called and reminded me to take meds every day. I also set my alarm clock. There was no problem.”* (B047, Female 25–29 years)

### Adolescence

We found that six YAPHIV in this study did not become aware of their HIV status until their early adolescent years, after they had been on ART for a while. They reported emotional responses followed by acceptance. Most reported that it did not affect their self-care or ART adherence because taking medication had already been a part of their routine. From the IDI, we did not find any positive social effects of HIV disclosure mentioned by study participants during their adolescent years.

### Negative social effects of HIV disclosure

Adolescents who grew up in orphanages that took care of many children with PHIV usually learned their serostatus from teachers and caregivers. However, some did not become aware of it until they were old enough to understand all the implications. A male participant described his feelings as startled when hearing that he had HIV. According to a friend who was older than him, being seropositive meant they could engage in high-risk behaviors without fear. He might have heard about HIV before but was unaware of his serostatus until that day. He recalled it as his first disclosure experience and self-acceptance. Increase risk taking behaviors was described by a male participant.

*“My heart was beating so hard when I first heard from an older friend that I had it (HIV) in my body. We lived together in an orphanage where everyone similarly took daily medicine. On that day I saw him with a lot of blood on his back. He had just gotten a tattoo. I asked him why he dared get a tattoo, as there was a risk of HIV infection. He said there was no more risk for him because all of us had HIV already.”* (B050, male 25–29 years)

HIV disclosure effect on family relationship was described by a male participant who stayed with his relatives stated that everyone in the family knew his status and he felt like they wished him to die rather than live into adulthood.

*“My relatives, I mean my older sister’s family with whom I stayed, knew my HIV status. They did not seem to care about it or me. I knew they wanted me to die, and they would get the funeral reimbursement.”* (B056, male 25–29 years)

Negative effect on schooling was mentioned by a female participant. She needed to quit school because of unintentional social disclosure, after she decided to tell someone who she thought would be discreet.

*“When I started the new vocational school, I went to my classroom teacher and told her that I would miss class to see the doctor for my HIV medication. After that she announced my name with this information in front of the morning class when all the students were together. My classmates looked at me as if I was disgusting. I felt very embarrassed and did not return to school after that.”* (B042, female 25–29 years)

### HIV non-disclosure

Many participants decided for HIV non-disclosure in their adolescent years, following self-acceptance of their HIV status. While participants had just learned their HIV status when they were adolescents, they were old enough to decide by themselves whether to tell anyone or to keep a secret. Two male participants reported that they felt nothing when learning of their status. However, neither disclosed their HIV status to others for different reasons. The former expressed a high level of anticipated stigma, while the latter perceived that it was unnecessary to disclose socially.

*“I started my meds at the age 13 after being hospitalized due to severe joint pain [HIV arthropathy]. I was not aware of HIV but took my meds regularly as I wanted to be symptom-free. Later my uncle disclosed my HIV status to me when I was in grade 8. I did not feel anything at that time. I think I was so young back then. [Did you talk to anyone?], …I just want to keep it to myself as I did not know what others would think about me.”* (B056, male 25–29 years)

*“My grandma disclosed my HIV status to me when I was in grade 10. I did not feel anything. All my relatives knew, but my schoolteachers and classmates were not aware of my HIV status. I have never told any of them. [Why didn’t you tell them?]. …they did not need to know.”* (B045, male 25–29 years)

A female participant described that she was a previously healthy child. When she started ART after hospitalization from a terrible cough, her mom only said that her immune was low. She was disclosed by her mom a year later. Back then, she was sad but kept taking her medication and decided not to tell anyone about it.

*“At that time, I was in grade 9. I felt sad but continued taking my meds and did not tell any friends about it.”* (B060, female 25–29 years)

Another male adolescent living in an orphanage where most residents were seronegative, said that he needed to hide medication from his peers and to avoid HIV-related conversations.

*“I lived in a male orphanage where most residents were HIV negative. None of my friends knew that I had it. I always hid my meds and took them when no one saw me. I didn’t know what their reaction would be if they knew. I had never wanted to mention it. When someone started talking about HIV, I would change the topic.”* (B053, male 21–24 years)

### Adulthood

When the study participants became adults, people always expected that they could achieve adult milestones, including getting a job, starting a sexual relationship, and having a family. Nevertheless, HIV disclosure remained a difficult decision to make. While many decided not to disclose to their partners or employers, a few did, and they found that it was not too bad or even better than they expected.

### Positive social effects of HIV disclosure

#### Perceived acceptance in relationships

Two male participants disclosed their status to their female partners and did not report either positive or negative responses, but simply acceptance.

*“My ex-girlfriend knew my HIV status as I told her while we were together. She did not express any feeling when she heard it. Everything between us was normal for almost 3 years until we separated at the beginning of this year.”* (B057, male 25–29 years)

*“When I told my girlfriend that I have HIV, she had no reaction and we continued being together. Then we had a kid, and we separated a year later. She still came to visit me and our boy who lived with me, and her HIV remained negative.”* (B041, male 21–24 years)

#### Perceived social support

A male participant disclosed his HIV status to his employer, and he felt glad for the acceptance and support received.

*“My boss teased me and asked if I had HIV. I said I did and showed him the paper from the hospital. He was stunned for a moment, then said ‘that’s OK, but you must take good care of yourself’. I could feel his care and support for me thereafter.”* (B056, male 25–29 years)

Another male participant said that he felt relieved that his girlfriend and her parents did not forbid their relationship after he disclosed his serostatus.

*“Very soon after we met, my girlfriend asked me about HIV as she had heard something from my colleagues. I told her about it, and she accepted my status without disgust. When her parents found out, they said it was OK, that today everyone has disease and there was nothing to worry about.”* (B052 male, 21–24 years)

### Negative social effects of HIV non-disclosure

As most study participants did not socially disclose their HIV status, we could not identify adverse social effects from the IDI. On the other hand, we identified an impact of non-disclosure on the quality of employment in a male participant who decided not to disclose his HIV status. A substantial social impact of non-disclosure was a lost employment opportunity. Some participants chose to sacrifice either work or social benefits to conceal their HIV status. A male participant avoided disclosure of his HIV status at his workplace as it seemed too scary for him, and he did not want to run the risk of being out of work. Instead, he decided to accept a non-permanent position which did not require HIV-testing.

*“I did not tell my employer about my HIV status. He offered me a permanent position, but I keep telling him that I want to continue working as a temporary employee. HIV testing is required to be a permanent employee, and I do not want to be tested.”* (B055, male 25–29 years)

Many participants did not disclose to their partners even while they were living together. They just did not mention the issue. A female participant has never told her husband about her HIV. His blood test was negative during the couple’s antenatal care, and she delivered two children without HIV transmission. They eventually separated due to other marital conflicts unrelated to HIV, but she never disclosed her HIV status although she kept thinking about it.

*“I have two kids, 10 and 11 years old. Their father, I mean my ex-husband, was never aware of my HIV status. During pregnancy, he went to the antenatal clinic with me and received a blood test. His HIV was negative, and he never asked me a word.”* (B060, female 25–29 years)

Another social effect of non-disclosure was avoiding sex in a relationship. A female participant mentioned her initiating a conversation about HIV with her boyfriend. He said no problem, but she still did not dare to disclose her status. She started her romantic relationship while keeping distance and avoiding having sex.

*“My boyfriend does not know my HIV status. I tried discussing a scenario asking him ‘what if I get HIV’. He said, ‘no problem, we can live together’. However, we have not talked about the fact that I really have it.”* (B047, female 25–29 years)

### A need for peer support

An emerging theme was identified in IDI. In their adulthood, study participants said they would like someone with seropositive status to talk to about HIV-related issues. However, in general, they did not have conversations with people they met in the HIV clinic other than their healthcare providers, though some made friends in their pediatric clinic. After transitioning out of pediatrics, they lost contact with those friends and felt isolated.

A female participant who did not disclose her status to anyone mentioned her loneliness and that one of her wishes was to have some friends her age with HIV with whom she could discuss whether to disclose status and how to deal with other HIV-related issues.

*“I want to talk to a friend who takes medicine like me. We might be able to understand each other more than general friends do. I have never known one in my life.”* (B047, female 25–29 years)

Another male participant expressed similar feeling of isolation while growing up. He recalled meeting some other boys in an HIV camp during childhood. However, they never met again after they transitioned out of pediatric care.

*“I went to a camp only a few times when I was young. I made some friends, but there was no phone or internet back then. We met by chance in the clinic several times. After we grew up and transitioned out, we did not see one another anymore. It would have been nice to have someone we can share our feelings like when we were down.”* (B055, male 25–29 years)

## Discussion

In this qualitative study of YAPHIV, we found that participants faced multiple challenges related to HIV disclosure while growing up. HIV disclosure was associated with strong emotions and various types of social effects including positive, negative, and neutral responses. YAPHIV have experienced dramatic changes in HIV diagnosis and treatment over the years. In the past, HIV was considered a dreadful disease. Without effective treatment, anyone who got the virus had to face an early death and social stigma due to the higher risk of transmission before ART. Now that there are effective treatments that increase lifespan and reduce transmission risk, having HIV has become an individual health condition, a condition that one can decide whether to tell anyone about. At the same time, as community members become better informed about the effectiveness of current interventions, HIV-related stigma and discrimination may gradually be reduced. This will also reduce the chances for negative consequences of disclosure.

The study findings indicated that social disclosure started very soon in the life of many individuals with PHIV. It often happened even before they were aware of the disease. The participants who grew up in communities where HIV was prevalent did not suffer from HIV status disclosure as a child. They were well-supported by neighbors and relatives. This result mirrored the findings in the U.S. Adolescent Medicine Trials Network for HIV/AIDS Interventions (ATN), where high perceived social support was reported in youth with HIV who disclosed their status (15). However, not all participants found out their serostatus when they were young, even though eventually the disclosure of the child’s HIV status to the child is essential. Despite the WHO recommendation (10), the decision to disclose serostatus to a child is not easy. The process can be planned and should consist of multiple steps rather than be a one-time event. A Thai study documented that the age of 9 years or older was a factor associated with caregivers’ readiness to disclose (21). In the Thai study, which provided readiness preparation training for caretakers of children with PHIV, found that only 71% of them were ready to disclose at the end of the one-year training (21). Although several barriers were identified including fear of stigma, fear of the child’s inability to keep their status secret, and emotional reactions from the child (22), our participants revealed that they did not suffer much from disclosure. They were generally able to manage their emotional reactions, could keep the secret, and continue their life. A previous study among adolescents’ caregivers in China revealed that reluctance to disclose was associated with concerns about stigma toward the children and families (23). However, in this study most participants mentioned that the disclosure was a neutral and non-emotive experience. Unlike adults with new HIV infection who frequently have feelings of guilt associated with the HIV diagnosis, children with PHIV looked at the disease from different perspectives. As a result, they might not have anticipated HIV -related stigma until they grew up and learned more about how other people felt and acted. Moreover, the reduced stigma might be related to the fact that they resided in small villages where everyone knew each other, and where many community members shared similar experiences of some family members, relatives, or friends dying of AIDS. On the other hand, some participants from more isolated families, i.e., without close connections and trust, decided to disclose to no one outside the family. In these cases, the child and his/her primary caregiver had to bear the burden of secrecy.

Many caregivers postponed HIV disclosure to their children due to the fear that disclosing would damage relationships in the family (24). Some of our participants were only made aware of their HIV status in early adolescence. Similarly, a UK study reported that disclosure happened past the age of 12 in half of their participants (25). Those adolescents mentioned that their subsequent feelings of isolation and distress due to the late disclosure was accompanied by a sense that they should keep their serostatus secret. An earlier disclosure experience facilitated participants’ decision to self-disclose later in life (26). When they were told of their serostatus, some participants did describe their feelings as sad or startled, but the knowledge did not affect their adherence or daily routines. The lack of negative impact might be due to a high level of support that these adolescents received from their caregivers. Other participants indicated that their feelings after disclosure were neutral. This reaction could be due to being in a phase of invincibility, which is a developmental period of adolescence in which they perceive themselves as special and believe they cannot be harmed by anything (27).

We learned that many of our participants did not disclose their status to friends due to fear of HIV-related stigmatization and discrimination. This confirmed the findings from the previous Thai study, in which 48% of adolescents disclosed their status to someone at the median age of 14.6 year (18). HIV disclosure to friends or romantic partners during adolescent years is particularly difficult as adolescents are very sensitive to rejection (28), and do not want to be perceived as different from others. Everyone must balance risk versus benefit following disclosure in their own context. After all, non-disclosure is a possible option, which is acceptable if it does not negatively affect self-care, ART adherence, and their mental well-being. The role of healthcare providers is to make sure that each adolescent has adequate HIV knowledge with sufficient self-efficacy and skills to disclose his/her HIV status if necessary. However, if they decide not to disclose, supporting them to adhere to treatment and minimize health risk behaviors is a feasible option under the treatment as prevention concept, “Undetectable is equal to Untransmittable” (U=U).

Upon entering young adulthood, most of them were seeking a permanent job and were starting their own family. HIV disclosure remained challenging due to anticipated stigma, fear of discrimination, and loss of support from their partner and others. While some participants disclosed their HIV status to others and received positive feedback, many were still reluctant to do so. Healthcare providers can help YAPHIV in exploring their reasons behind their decision and help them manage their social disclosure plan.

Most of our participants decided not to disclose their HIV status to their partner, while trying to maintain treatment adherence to protect their partner. This reluctance to disclose might be due to their young age when starting a relationship. Although the frequency of HIV disclosure by youth with HIV to main partners was reported as higher than the disclosure to casual partners (29), one study documented that adolescents were less likely to disclose to main partners when compared with older adults (30). Two studies in the US and the UK reported both positive and negative consequences of HIV disclosure on sexual relationships including receiving support, rejection, and struggling (31, 32). Adolescents and young adults should be supported to find the balance between the benefits and risks of HIV disclosure to partners. HIV disclosure could increase physical protection from HIV transmission while non-disclosure could offer emotional protection from being rejected (32). Healthcare providers and counselors could adopt the treatment as prevention concept and become more comfortable in supporting their clients’ decision to defer disclosure until the proper time, while emphasizing the need for strict adherence to ART. Non-disclosure might lead to missing opportunities to enjoy social support that could buffer their emotional distress while living with HIV (33). Monitoring and continuing to support clients’ autonomy is required as a part of HIV care.

## Study limitations

The study’s limitations included the fact that we might not have included YAPHIV with extremely negative consequences after HIV disclosure as they might not have been willing to share their painful experiences. A second limitation is that the social context has changed, and that these findings might not be applied to children and adolescents with PHIV today. With widely available treatment, HIV is not a fatal dreadful disease as it was, but HIV-related stigma still presents. As not many people in the community have AIDS and HIV infection is more concentrated in the key population, HIV disclosure can be either easier or more difficult than it was in the past. Ongoing HIV disclosure research in different populations might be beneficial. Those might include adolescents who were born in a digital environment with easily accessible health information, those living with their biological parents, and HIV-exposed uninfected youth. These populations might have different disclosure stories and need different types of support.

## Conclusion

The strength of this study is that we have documented YAPHIV sharing real experiences from their memories. It would help us as healthcare providers to understand our clients and their real-world challenges. YAPHIV are still facing ongoing challenges associated with HIV disclosure to others while growing up and moving through life. Apart from regular HIV care, health education, and social support, healthcare providers could also play a role in providing their clients with disclosure self-efficacy and coping skills to meet their needs and achieve adult milestones seamlessly.

## Data availability statement

The raw data supporting the conclusions of this article will be made available by the authors, without undue reservation.

## Ethics statement

The studies involving human participants were reviewed and approved by the Human Experimentation Committee, Office of Research Ethics of the Research Institute for Health Sciences, Chiang Mai University. The patients/participants provided their written informed consent to participate in this study.

## Author contributions

LA: overall study design, data analysis, and writing manuscript. AT: study supervision, data analysis, guidance on writing, review, and approve manuscript. CD, SM, AS, PM, and WC: conduct study, data analysis, and review manuscript. All authors contributed to the article and approved the submitted version.

## Conflict of interest

The authors declare that the research was conducted in the absence of any commercial or financial relationships that could be construed as a potential conflict of interest.

## Publisher’s note

All claims expressed in this article are solely those of the authors and do not necessarily represent those of their affiliated organizations, or those of the publisher, the editors and the reviewers. Any product that may be evaluated in this article, or claim that may be made by its manufacturer, is not guaranteed or endorsed by the publisher.

## Footnotes

^1^ www.dedoose.com
